# Exploring the climate change concerns of striped catfish producers in the Mekong Delta, Vietnam

**DOI:** 10.1186/s40064-015-0822-0

**Published:** 2015-02-01

**Authors:** Anh Lam Nguyen, Minh Hoang Truong, Johan AJ Verreth, Rik Leemans, Roel H Bosma, Sena S De Silva

**Affiliations:** Institute of Aquaculture, Nha Trang University, 2 Nguyen Dinh Chieu Str., Nha Trang City, Vietnam; College of Aquaculture and fisheries, Campus II, 3/2 Street, Ninh Kieu District Can Tho City, Viet Nam; Aquaculture and Fisheries Group, Wageningen University, De Elst 1, 6708 WD Wageningen, The Netherlands; Environmental Systems Analysis Group, Wageningen University, Droevendaals Steeg 3, 6708 PB Wageningen, The Netherlands; School of Life & Environmental Sciences, Deakin University, Warrnambool, VIC 3280 Australia

**Keywords:** Mekong Delta, Pangasius farmer, Climate change, Perception, Adaptation

## Abstract

This study investigated the perceptions on and adaptations to climate change impacts of 235 pangasius farmers in the Mekong Delta, Vietnam. Data were collected using semi-structured household surveys in six provinces, from three regions along the Mekong river branches. A Chi-Square test was used to determine the association between variables, and a logit regression model was employed to identify factors correlated with farmer’s perception and adaptation. Less than half of respondents were concerned about climate change and sought suitable adaptation measures to alleviate its impacts. Improving information on climate change and introducing early warning systems could improve the adaptive capacity of pangasius farmers, in particularly for those farmers, who were not concerned yet. Farmers relied strongly on technical support from government agencies, but farmers in the coastal provinces did not express the need for training by these institutions. This contrasting result requires further assessment of the effectiveness of adaptation measures such as breeding salinity tolerant pangasius.

**Electronic supplementary material:**

The online version of this article (doi:10.1186/s40064-015-0822-0) contains supplementary material, which is available to authorized users.

## Introduction

The pangasius (*Pangasianodon hypophthalmus* Sauvage 1878, Striped catfish in English or tra in Vietnamese) farming sector, which originated in the Mekong Delta, is among the fastest growing aquaculture sectors during the past two decades in the world. The Mekong Delta (8°33’- 10°55’N; 104°30’-106°50’E) is ranked among the deltas that will be most affected by climate change (Dasgupta et al. 2007; Syvitski et al. 2009). In Vietnam, from 1958 to 2007, the reported increase in temperature is about 0.5 to 0.7°C, and average rainfall decreased by about 2% (MONRE Ministry of Natural Resources and Enivironment 2009). Due to sea level rise, experts have projected negative impacts of increasing water level and salinity intrusion on dams and infrastructure (Hoa et al. 2007, 2008), rice production (Wassmann et al. 2004; Khang et al. 2008), or the location of pangasius farms (Anh et al. 2014) in Vietnam.

Adaptation is widely recognized as a vital component of any policy response to climate change (Gbetibouo 2009). Smit et al. (2001) defined adaptation as any adjustment in natural or human systems that takes place in response to actual or expected impacts of climate change, and which are intended either to moderate harm, or to exploit beneficial opportunities. To investigate the factors influencing farmer’s perception of and adaptation to climate change, research teams used either descriptive statistics (Mertz et al. 2009; Tambo and Abdoulaye 2013) or logit regression models (Deressa et al. 2008; Apata et al. 2009; Gbetibouo 2009; Fatuase and Ajibefun 2013). The above studies concluded that most farmers had noticed changes in climate and adjusted farm practices, but lacked precise information on climate change and suitable adaptation measures. Access to credit limited the farmer’s adaptation possibilities also (Gbetibouo 2009; Fatuase and Ajibefun 2013).

In the Mekong Delta, climate change is predicted to increase salinity levels and extend its effects in the four coastal and two central provinces and will increase the risk of flooding in the upstream and central provinces (Anh et al. 2014). Consequently, livelihoods of pangasius farmers operating in the lower reaches of the two main branches of the river may be threatened. Kam et al. (2012) showed that catfish farmers’ operations are vulnerable to climate change. The projected benefits of the inland pangasius farms remain positive when climate change is ignored but disappear when climate change is considered. The farmer’s autonomous adaptation measures (such as increasing height of pond dykes) are costly, and the benefits of coastal pangasius farms will be halved under the climate change scenario explored by Kam et al. (2012).

The farmers’ skills and responsiveness can positively influence these benefits but this may strongly depend on their awareness of climate change and the possible adaptation choices (Gbetibouo 2009). The pangasius farmers’ awareness and choices are, however, poorly known. As such knowledge is crucial for the effective adoption of adaptation strategies, this study investigates (1) the pangasius farmers’ perception of climate change and its impacts, (2) the adaptations measures adopted by them, and (3) the relationship between perceived impacts and measures with farm characteristics.

To assess to awareness on, perception of and adaptation to climate change of pangasius farmers, and the factors influencing both we collected data in six provinces of the Mekong Delta. The research questions, the study area and a brief history of the pangasius system, and methods of data collection and analysis are presented in section 2. After having presented the results we link our findings to other research in the discussion before concluding.

## Materials and methods

Relating to pangasius farmers this study aims to answer the following research question : Are they aware of Climate Change. Did they perceive Climate Change impact? Did they adapt or do they plan to adapt to Climate Change. Which are their implemented or preferred adaptation measures, and would they need information or support to implement these measures?

### Short overview of the Pangasius farming sector and the study area

The development of the pangasius sector, together with associated controversies such as criticism on environmental, social and safety credentials, have been well documented (Bosma et al. 2009; Phan et al. 2009; Bui et al. 2010; De Silva et al. 2010; Nguyen 2010; De Silva and Phuong 2011; Little et al. 2012). In 2012, the sector produced 1.2 million tons of fish in a pond area of 5.600 ha and exported the processed pangasius, mainly in the form of fillet, to over 142 countries with a value of 1.7 billion US$ (VASEP 2013).

The industry started with cage culture of caught fries of *Pangasius bocourti* (De Silva and Phuong 2011) in the upstream part of the main branches of the Mekong river that runs through An Giang, Dong Thap and Can Tho provinces. The land-based pond culture of Striped catfish started with fries recruited in the court’s pond after retrieval of the annual floods. Land-based pond culture took a hold, particularly after the successful artificial propagation of Striped catfish that ensured seed stock supplies (Nguyen 2009; De Silva and Phuong 2011; Bui et al. 2013). After farmers and research institutions identified an optimal intensive practice, land-based pond culture the industry expanded downstream in the Vinh Long, Tra Vinh, Soc Trang and Ben Tre provinces. At present the farming system entails independent small-scale farmers and holdings, both delivering their fish to processing companies, as well as fully integrated companies with own feed-mills, ponds and processing facilities.

**Figure 1:**
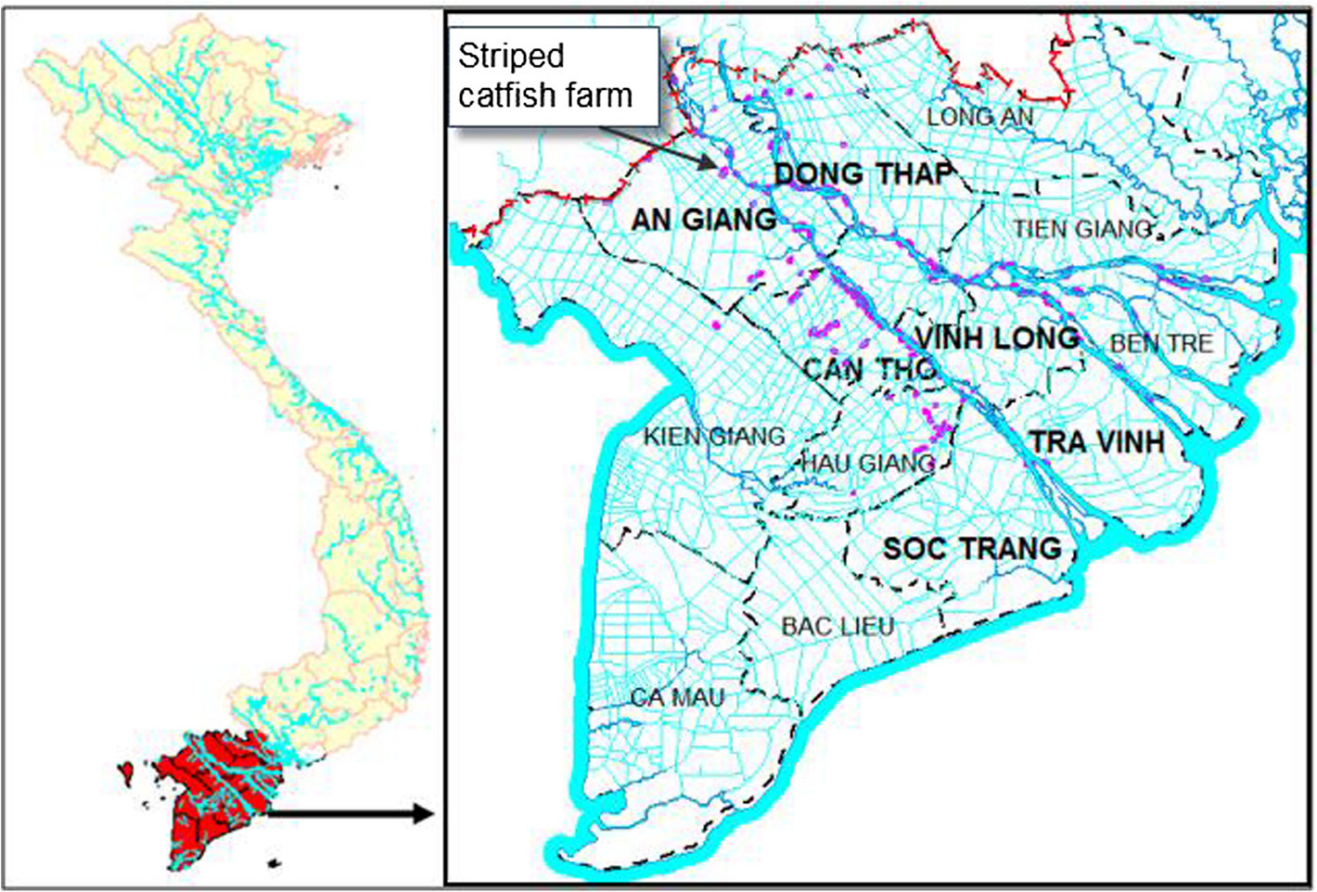
**Administrative map of the Mekong delta with the locations of the striped catfish farms in 2009 (adapted from Anh et al.** 2014 **).**

The study was conducted in the six provinces of Mekong Delta (8°33’- 10°55’N; 104°30’-106°50’E) where Striped catfish farming is most prevalent: An Giang, Can Tho, Dong Thap, Soc Trang, Vinh Long and Tra Vinh (Figure 1). The six provinces were categorized into three production regions: upstream (An Giang and Dong Thap), mid-stream (Vinh Long and Can Tho) and downstream (Tra Vinh and Soc Trang).

### Data collection

To answer these questions we collected data on variables relating to farm and farmer characteristics, and on the farmer’s actions, perceptions and suggestions regarding climate change. Among others the farmers were asked whether they observed any phenomenon of climate change in the last ten years and whether they were concerned about its impact. The adaptation and mitigation options suggested to the respondents: change farming practice, change target species, or abandon aquaculture, were based upon the answers given during the testing of the questionaires. A draft questionnaire covering general information on the farmer’s household, costs of different inputs, production and income, as well as the farmer’s perceptions and strategies to climate change induced changes, was developed by experienced socio-economists and climate-change scientists of the Aquaclimate project (Nagothu et al. 2009). The draft questionnaire was tested, modified and finalized for conducting the detailed survey. The survey was conducted through face to face interviews of farmers by trained personnel with past experience on conducting such surveys. Each interview was conducted by two interviewers.

We used proportional random sampling to select farms to interview in each province. The number of farms to survey per province was determined by the overall farm frequency distribution. The respondents were either the farm owners or the farm managers in case of the farm being part of a larger company. Data from five provinces (Dong Thap, Can Tho, Vinh Long, Soc Trang and Tra Vinh) were collected in the framework of the Aqua-Climate project in 2009. In 2011 the data for An Giang were supplemented to cover the six provinces and balance the sample. We did not include Ben Tre province because most ponds recently became owned by two companies only, and present farm managers had little historical perspective on the impacts of climate related events.

The survey data were entered into a database developed for the purpose in MS Excel. Data from 235 farms were available consisting of 45, 53, 25, 82, 15, and 15 farms from An Giang, Dong Thap, Vinh Long, Can Tho, Soc Trang and Tra Vinh provinces, respectively.

### Data analysis model

The analysis in this paper focuses on the perceptions and strategies to climate change induced changes. The resulting survey variables were ordinal or categorical. To understand the association between these, different statistical methods were used. The Chi-Square test was used to determine the extent of association between the variables. To examine the factors which influence the farmer perceptions of and adaptations to climate change, a logit regression model was employed (Deressa et al. 2008; Apata et al. 2009; Gbetibouo 2009; and Fatuase and Ajibefun 2013).

The standard form of the logit regression model is:

1 $$ ln\left(\frac{p}{1\kern0.5em -\kern0.5em p}\right)\kern0.5em =\kern0.5em {\beta}_0\kern0.5em +\kern0.5em {\varSigma}_{i=1}^n{\beta}_1{X}_1\kern0.5em +\kern0.5em \varepsilon $$

Where *p* is the probability that the dependent variable value is 1 and *(1-p)* is probability that dependent variable value is 0; *β*_*i*_ is the parameter estimate for the independent variable *X*_*i*_ , and *i* is the variable number; and *ε* is the error term. Marginal effects of the explanatory variables from equation (1) are estimated according to Green (2000). The marginal effects are functions of the probability itself and measure the expected change in probability of a particular choice being made with respect to a unit change in an independent variable from the mean. All the statistical analyses were done using R software package.

## Results

### Respondants’ and farm’ characteristics

The interviewed farmer’s average age was 45 year (Table 1). The older farmers lived in the downstream region. Their aquaculture experience ranged from 1 to 20 years with an average of 5.5 years. The average aquaculture experience decreased from 5.7 years to 4.3 years from upstream to downstream.

**Table 1:** **The mean values and standard deviations (±SD) of the main characteristics of the surveyed pangasius farmers for the aggregated six provinces and for the three regions**

	**All provinces (235 farms)**		**Upstream provinces An Giang, Dong Thap (98 farms)**		**Mid-stream provinces Vinh Long, Can Tho (107 farms)**		**Downstream provinces Soc Trang and Tra Vinh (30 farms)**	
	**Mean**	**±SD**	**Mean**	**±SD**	**Mean**	**±SD**	**Mean**	**±SD**
Age (year)	44.5	10.4	44.1	9.3	44.1	11.1	47.4	10.6
Total ponds area (ha)	0.8	0.6	0.8	0.6	0.8	0.7	0.4	0.4
Education level of respondents*	2.4	0.8	2.6	0.7	2.4	0.8	1.8	0.9
Land ownership (1: owner and 0: otherwise)	0.7	0.5	0.8	0.4	0.6	0.5	0.8	0.4
Culture experience (year)	5.5	3.1	5.7	3.3	5.7	3.2	4.3	1.6
Contribution of pangasius farming to income (%)	82.2	24.2	87.1	17.8	84.5	23.8	58	29.8

*1 = primary school; 2 = secondary school; 3 = high school; 4 = bachelor degree.

The educational level of respondents varied from primary school (level 1) to bachelor degree (level 4) with an average higher than level 2 (secondary school). Among them, 15% finished primary school, 39% had a secondary school certificate, 37% graduated from high school and 8% got a bachelor degree. The educational level was highest in the upstream and lowest in the downstream regions.

The total pond’ area per farm ranged from 0.08 ha to 3.8 ha with an average of 0.8 ha. The average total pond area in the downstream region was only half of that in up and mid-stream regions. In the up- and downstream region, 80% of the respondents were land owners whereas only 60% of the farmers in the mid-stream region owned land.

The pangasius revenues contributed 87% to the income of the farm-households upstream, 85% in mid-stream, and only 58% in the downstream region. The downstream farms acquired 1% of their income from rice cropping, 3% from services, 11% from other activities, and 27% from orchards.

### Farmer’s perceptions on climate change

Table 2 presents the awareness of interviewed farmers about climate change. Four categories of climate change impact were proposed to the farmers: stronger flood in rainy season, serious drought in dry season, fluctuated temperature, or expanded salinity intrusion. Less than half of the respondents (43%) had observed the climate change occurence and slightly more (45%) were concerned about is impact. However, none of the downstream farmers had observed or was concerned about climate change, while around 70% of the upstream and 30% of the midsteam farmers had observed and were concerned. The more frequent farmers had experienced extreme weather events the higher their concern. Surprisingly, no pangasius farmers in the coastal provinces appeared to have observed, nor were concerned abourt climate change. The number of upstream farmers observing and concerned about climate change was highest at 68% and 74.5%, respectively, and these numbers were average for mid-stream farmers: 31% for both responses.

**Table 2:** **The perception on Climate Change (CC) and on Climate Change Impacts (CCI) of the interviewed pangasius farmers (% agreed)**

	**Observed climate change**	**Concern about CC**	**CCI in rainy season**	**CCI in dry season**	**CCI on temperature**	**CCI on salinity intrusion**
All 6 provinces (n = 235)	42.5	45.0	40.5	66.0	60.5	16.5
Upstream provinces (n = 98)	68	75	55	45	46	11
Mid-stream provinces (n = 107)	31	31	33	81	75	0
Downstream provinces (n = 30)	0	0	20	80	57	93

More than half of the respondents in upstream area (55%) thought climate change will lead to stronger flooding in the rainy season, whereas only about one-third of the respondents in mid-stream and 20% downstream thought likewise. Inversely, about 80% of the respondents in the mid-stream and down-stream regions expected impact of climate change in the dry season. Less than half of the upstream farmers feared impacts in the dry season.

A change of temperature caused by climate change was expected by respondents in the whole delta, but most in the mid-stream region (75%) and lowest upstream (46%). Almost all downstream respondents (93%) feared an increase of salinity intrusion due to climate change, whereas upstream only 11% and in the mid-stream region none shared this opinion.

The chi-square test confirmed the interacttion between farm location (region) and the farmer’s perceptions (p < 0.001). However, the education level did not significantly affect their awareness on climate change (p > 0.05).

### Farmer’s preferences for adaptation

Only 47% of the respondents considered to adapt to climate change impact by using one of the proposed adaptation measures whereas the remainders did not know. Among the respondents intending to adapt¸ close to 40% preferred to change farming practice, while 5% would change the cultured fish species and another 5% would abandon aquaculture (Table 3). Nearly 55% of respondents upstream thought to change farming practice, while about 30% in the mid- and down-stream regions would do so. The number of farmers expecting to change the target species was slightly higher up-stream than mid-stream, while down-stream none expected to shift target species. The latter contrasts with the option to abandon aquaculture: 17% of the farmers downstream would prefer and select this option.

**Table 3:** **The farmer’s preference among three adaptation options (%)**

	**Change farming practice**	**Change species**	**Abandon aquaculture**
All six provinces	39.5	5.0	5.5
Upstream provinces	55	8	5
Mid-stream provinces	28	3	3
Downstream provinces	30	0	17

The region variables were highly associated with the adaptation options ‘change farming practice’ and ‘change species’ (p < 0.001), and ‘abandon fish farming’ (p < 0.05). The education level was associated with the expectation to adapt through abandoning aquaculture (p < 0.05).

### Farmer suggestions

More than half of the interviewed farmers desired assistance to adapt efficiently to climate change impacts. They preferred to receive information on lessons learned or financial support from their friends or family (Table 4). The support of government institutions, local government and private sector would be less appreciated, but some regional differences were apparent.

**Table 4:** **Farmers’ suggestions for sources of support to adaptation of climate change, and the focus of this support (% agreed with each suggestion)**

	**Source of support**				**Type of support**		
	**Government institution**	**Local government**	**Private sector**	**Friend/family**	**Technical**	**Financial**	**Training**
All 6 provinces	10.2	4.7	6.0	17.0	53.2	20.9	19.6
Upstream provinces	14.3	9.2	5.1	22.4	53.1	24.5	39.8
Mid-stream provinces	8.4	1.9	8.4	15.9	52.3	21.5	6.5
Downstream provinces	3.3	0.0	0.0	3.3	56.7	6.7	0.0

More than half of the respondents (53%) suggested technical support from government agencies to develop proper adaptation measures, for example by providing adapted seed and practical advices for improvement. More than 20% of respondents in up- and mid-stream wanted to get a loan with appropriate interest rates from the government bank whereas only 7% of the downstream farmers desired this support. Attending training provided by the fisheries extension service may support them to adapt efficiently according to approximately 40% of the up-stream respondents, but only 6.5% in mid-stream and none in down-stream thought this would help (Table 4).

### Factors influencing farmer awareness

In general the variation explained by one single parameter of farmer perceptions and expectations regarding climate change and its impacts remained below 0.1 and the total explained variation below 0.2 (Table 5). Some factors shifted to negative or positive when the analysis was done per region. The age and education level of farmers, and the income from pangasius aquaculture affected their perceptions and concerns about climate change slightly positively (Table 5). Ownership of the farm positively influenced the farmer’s fear for salinity intrusion, but the influence of the contribution of pangasius aquaculture to household income factor was inverse. Aquaculture experience only affected farmer’s expectation regarding the impact on temperature (p < 5%). Only ownership of the farm significantly (p < 5%) affected the farmer opinions on the option to mitigate the effect of climate change by changing farming practice (Table 5).

**Table 5:** **Marginal effects of farmer’s characteristics on their perceptions of and adaptation to climate change through correlation coefficients (as determined by the logit model)**

	**Observed climate change**	**Concern about climate change**	**Impact of climate change expected on:**				**Preferred adaptation**		
**Parameters**			**Flood**	**Drought**	**Temperature**	**Salinity intrusion**	**Change farming practice**	**Change species**	**Abandon aquaculture**
	**Coeff.**	**Coeff.**	**Coeff.**	**Coeff.**	**Coeff.**	**Coeff.**	**Coeff.**	**Coeff.**	**Coeff.**
Age of farmer	0.010^**^	0.007^*^	−0.006	0.002	0.001	−0.002	−0.005	−0.001	0.001
Total ponds area	0.056	0.080	0.041	−0.060	0.006	−0.016	−0.068	0.030	0.035
Education level	0.075	0.077^*^	0.064	−0.048	−0.021	−0.046	0.063	0.000	0.019
Ownership	0.063	0.015	−0.034	−0.012	0.025	0.127^**^	0.143^*^	0.056	0.060
Income from aquaculture	0.003^*^	0.003	−0.002	−0.001	0.001	−0.003^***^	−0.001	0.000	0.000
Aquaculture experience	0.001	0.006	0.005	0.005	0.030^*^	−0.011	0.004	0.004	−0.006

Note: ***significant at 0.1%, ^**^significant at 1%, ^*^significant at 5%

## Discussion

In agreement with other studies (Deressa et al. 2008; Apata et al. 2009; Gbetibouo 2009; Fatuase and Ajibefun 2013), pangasius farmers in the Mekong Delta were aware of the change in temperature (60% of respondents) and the occurence of droughts (66% of respondents). The farmer’s perceptions on climate change impacts were influenced by characteristics of both farmer and farm. The farmers’ age had a positive relationship with climate change awareness and concern. Older farmers have experienced more extreme weather events and may have observed changes so that they became more aware of climate change and more concerned about its negative impacts compared to younger farmers. The positive effect of education level on concern about climate change impacts may be due to the fact that educated farmers gained knowledge on climate change and thus expected more negative influences caused by climate change occurrence than less educated farmers. In our study, 75% of the upstream farmers were concerned about climate change phenomena, because they had experienced extreme flooding. Downstream farmers were not concerned but had adapted their farming system to salinity. Farm owners have a higher likelihood of expecting salinity intrusion to impact negatively the culture of fresh water species like pangasius, but having a higher part of the income from pangasius aquaculture decreased the fear for salinity. Apparently the ones making a benefit, notwithstanding the salinity intrusion, know how to deal with the problem effectively, even if they were more aware of climate change and had higher concerns on its impacts. They also invested more in measures to reduce the impacts of extreme weather induced by climate change.

Across the regions, about 50% of the farmers considered adaptation measures. Most among these farmers chose to change farming practices, for example, increase the height of dykes, improve water quality or decrease the stocking density. Change of farming practice included, for example, increase the height of dykes, improve water quality and decrease the stocking density. All these measures are costly. Seven to eight times less farmers expected to change species or to abandon aquaculture. Changing species requires acquiring new knowledge and skills, and refreshing networks. Farmers owning the land were more inclined to change farming practice, inversely abandoning is an option for those renting the land or pond. The latter leaves the land owner a deep pond which has no other agriculture use. Part of the farms in the upstream region were located within areas with large flood protection dikes which reduces the need for autonomous. A farmer in the Chau Thanh district of the An Giang province stated: “I don’t need to increase my pond-dyke because the government’s dyke is high enough”.

The difference in length of experience with pangasius culture between downstream and the more upstream provinces is likely related to the history of development of pangasius farming along the Mekong branches. The development of pangasius farming in the Mekong Delta spread out along the Mekong river branches from upstream to downstream providing the farmers in upstream area a longer aquaculture experience than those downstream. The downstream farmers are less dependent on the pangasius revenue to household income than those in the mid- and up-stream regions (58% compared to 86%). This might be the result from lessons learned upstream. Pangasius farming in the downstream region started after the price dip due to the USA import ban (Tung et al. 2004), and the fluctuation of the pangasius market price stimulated downstream farmers to maintain other components of their farm, such as the high yielding orchards.

Nevertheless, due to our qualitative method of the semi-structure household survey, combined with the gradual impacts of climate change and the strong effects of extreme weather events on farmer perceptions (MRC 2012; Anh et al. 2014), the responses of pangasius farmers were influenced by many factors such as farm location, experience and education. Our results support growing evidence that vulnerability, as well as the capacity to adapt, may differ from one region to another, and within a region there may be differences between communities or individuals (Gallopin 2006). For instance, 11% of the respondents in the upstream region had never faced salinity intrusion, but were still afraid about its possible occurrence. Thus, farmer responses may be biased causing uncertainty in the statistical analysis.

The projected impacts of sea level rise caused by climate change on pangasius farming locations by Anh et al. (2014), showed pangasius farmers in coastal province will seasonally face increasing salinity intrusion for longer periods. The downstream farmers thought training from extension services would not enforce their capacity to adapt. Fish farmers in Delta State of Nigeria perceived low yields of fish culture due to adverse impacts of climate change, and the number of fish ponds, income and extent of knowledge positively affected farmers’ perception on climate change impact (Aphunu and Nwabeze 2012). About 85% of the fish farmers stated to need more information on climate change. Farmers in the Nigerian savanna had limited capacity to adapt because they lacked information on climate change and on suitable adaptation measures (Tambo and Abdoulaye 2013). The low trust in training by farmers in the downstream region reflected the low government priority on pangasius aquaculture in coastal provinces as well as the lack of specific pangasius extension officers.

Abandoning pangasius farming or changing species will have consequences on the value chain and its rates of return and employment. Therefore future strategies to increase resilience of pangasius farmers to climate change should focus on improving farmers’ knowledge and skills, not only on technical aspects but also on awareness about climate variability and measures to address the impacts.

Adaptation measures such as increasing the height of dykes and improving water quality are costly. According to Kam et al. (2012) the compounding effect of climate change will increase additional cost of adaptation for pangasius farmers and further reduce their profit margins. This is without accounting the effects government protection dikes on biodiversity and rice productivity (Lebel et al. 2009; Marchand et al. 2014). The latter authors suggested adaptation through reducing cost of electricity and fuel by technical improvements, including the selection for higher salinity tolerant catfish species, the replacement with salinity tolerant species, or the change in farming practices. More than half of the respondents expected support from various government agencies for their adaptation, in particular regarding the protection dikes (MARD Ministry of Agriculture and rural Development 2008) and zoning of fish farming according to species. However the farmers don’t fear salinity intrusion and thus the efficiency of introducing a salinity tolerant pangasius breed for climate change adaptation in the downstream region, as suggested by De Silva and Phuong (2011), needs to be evaluated in depth.

## Conclusion

Most pangasius farmers in the upstream region of the Mekong Delta had experienced reduced financial benefits due to extreme flooding. Downstream, in the coastal provinces, from the start farmers were confronted with seasonally high and gradually increasing salinity levels. They have taken measures, such as increasing height of dyke, or decreasing the number of stockings, or stocking larger fish tolerating higher salinity levels, to adapt to these impacts. Most farmers, however, will eventually feel the gradually increasing effects of climate change. Less than half of the respondents were, however, concerned about these climate change impacts and sought for suitable adaptation measures.

Farmer’s education level, the age and aquaculture experience, the dependency on income from pangasius farming and whether they own the land increase their awareness of and concern about climate change impacts. At present the respondents mainly count on support and lessons learned from friends and family to realize adaptation measures. Respondent’s high expectation on technical support from government agencies contrasts with their low expectations from training in particular in the coastal provinces. Alike in other countries, building more awareness on climate change is required to improve adaptation capacity for pangasius farmers.
